# Improved Methods for Capture, Extraction, and Quantitative Assay of Environmental DNA from Asian Bigheaded Carp (*Hypophthalmichthys* spp.)

**DOI:** 10.1371/journal.pone.0114329

**Published:** 2014-12-04

**Authors:** Cameron R. Turner, Derryl J. Miller, Kathryn J. Coyne, Joel Corush

**Affiliations:** 1 Department of Biological Sciences, University of Notre Dame, Notre Dame, Indiana, United States of America; 2 College of Earth, Ocean, and Environment, University of Delaware, Lewes, Delaware, United States of America; Temasek Life Sciences Laboratory, Singapore

## Abstract

Indirect, non-invasive detection of rare aquatic macrofauna using aqueous environmental DNA (eDNA) is a relatively new approach to population and biodiversity monitoring. As such, the sensitivity of monitoring results to different methods of eDNA capture, extraction, and detection is being investigated in many ecosystems and species. One of the first and largest conservation programs with eDNA-based monitoring as a central instrument focuses on Asian bigheaded carp (*Hypophthalmichthys* spp.), an invasive fish spreading toward the Laurentian Great Lakes. However, the standard eDNA methods of this program have not advanced since their development in 2010. We developed new, quantitative, and more cost-effective methods and tested them against the standard protocols. In laboratory testing, our new quantitative PCR (qPCR) assay for bigheaded carp eDNA was one to two orders of magnitude more sensitive than the existing endpoint PCR assays. When applied to eDNA samples from an experimental pond containing bigheaded carp, the qPCR assay produced a detection probability of 94.8% compared to 4.2% for the endpoint PCR assays. Also, the eDNA capture and extraction method we adapted from aquatic microbiology yielded five times more bigheaded carp eDNA from the experimental pond than the standard method, at a per sample cost over forty times lower. Our new, more sensitive assay provides a quantitative tool for eDNA-based monitoring of bigheaded carp, and the higher-yielding eDNA capture and extraction method we describe can be used for eDNA-based monitoring of any aquatic species.

## Introduction

Environmental DNA (eDNA) is DNA extracted from environmental samples (e.g., soil, water, air) without first isolating the target organisms or their parts [1], [2]. The concept and the term both originate from microbiology [3] where the target DNA in environmental samples is from abundant live and dead microbes. In contrast, macrobial eDNA is the DNA of large organisms such as animals or plants that occurs in environmental samples. Although macrobial eDNA has been studied since 1991 in fields such as human forensics [4], agricultural transgenics [5], paleogenetics [6], and fecal pollution source tracking [7], it was only in 2008 that it was first used for aquatic macrofauna [8]. Aqueous macrobial eDNA has garnered particular interest [9], [10] as a simple and sensitive way to detect rare aquatic macrofauna such as invasive or endangered vertebrates and invertebrates [11]–[20]. In comparison, direct observation of rare organisms often has low detection probability [21], limited seasons [22], high costs [23], and increased risk of harming sensitive species [24].

One of the first and largest conservation programs with eDNA-based monitoring as a central instrument is focused on Asian bigheaded carp (*Hypophthalmichthys* spp., hereafter bigheaded carp) [25]–[28]. Bigheaded carp were imported to North America as two separate species, Bighead Carp (*Hypophthalmichthys nobilis*) and Silver Carp (*Hypophthalmichthys molitrix*). However, in the Mississippi River basin *Hypophthalmichthys* hybridization is widespread, including fertile post-F_1_ hybrids and F_1_ hybrid frequency estimates as high as 73% for the *H. molitrix* morphotype [29]–[31]. This hybrid swarm may be developing into a new species complex [30] as the genus expands its range northward [32], [33]. These large planktivorous fish threaten fisheries due to their dietary overlap with native filter feeders [34] and their tendency to reach high abundance and biomass in their invaded range [35]. These characteristics have implicated bigheaded carp in the decline of at least two commercially important fish species in the Mississippi basin, gizzard shad (*Dorosoma cepedianum*) and bigmouth buffalo (*Ictiobus cyprinellus*) [36]. Recent analyses predict that small introductions of bigheaded carp could become established [37] and cause significant ecological and economic harm in many coastal embayments, wetlands, and tributaries of the Laurentian Great Lakes [38], [39].

eDNA-based monitoring to provide early detection of bigheaded carp is a central instrument in the ongoing, binational effort to prevent their establishment in the Great Lakes [25], [26], but the methods currently used for capturing, extracting, and assaying bigheaded carp eDNA have not advanced since their development in 2010 [25], [40]. Their continued application has revealed inconsistent performance, including failure to detect *H. nobilis* at a site in the Mississippi River where they are considered abundant [41]. Our objective was to develop a set of tools for eDNA-based monitoring of bigheaded carp that are more effective and affordable than the current standard protocol. We present new methods that increase sensitivity and objectivity, decrease cost, and add quantitative information compared to existing protocols. These consist of a quantitative polymerase chain reaction (qPCR) assay specific to bigheaded carp eDNA, a polycarbonate track-etched (PCTE) filter membrane for capturing eDNA, and a cetyl trimethyl ammonium bromide (CTAB) DNA extraction protocol. We compare the performance of new and old methods using paired samples from an experimental pond containing bigheaded carp.

## Materials and Methods

### Ethics Statement

No permits were required for sampling at any of the sites in this study, however several were privately owned and required permission for sampling, as noted in Table S1. Field sampling did not involve any endangered or protected species, and sampling locations are provided in Table S1. No animal welfare or animal use and care protocols were required for this study, as no vertebrate animals were directly utilized (only environmental samples were collected and we did not directly house or manipulate any animals).

### Adaptation of microbiology methods to capture and extract aqueous macrobial eDNA

For decades, environmental microbiologists have developed, refined, and compared methods to capture and extract DNA from environmental samples [42]. We selected a widely used approach from aquatic microbiology wherein water samples are filtered through PCTE membranes and the filter retentate is extracted using a CTAB protocol wherein chloroform chemically dissolves the PCTE filter [43], [44]. We chose PCTE membranes (47 mm diameter, GE Osmonics, sold by Barney Corporation, Hilliard, Ohio, USA) with a 10 µm pore size because in side-by-side trials one PCTE membrane allowed filtration of 2 L of water in approximately the same amount of time (∼10 min) required to filter 2 L of pond water through one 1.5 µm pore size glass fiber (GF) filter (47 mm diameter, grade 934-AH, Whatman, GE Healthcare Life Sciences, Piscataway, New Jersey, USA). The 934-AH GF filter and 2 L water volume are specified in the current standard operating procedure for eDNA-based monitoring of bigheaded carp [25], [40]. Our CTAB DNA extraction protocol (Protocol S1) differs from that of Coyne et al. [43], [44] only in the absence of β-mercaptoethanol from the CTAB buffer, which we removed for convenience because it produces a strong disagreeable odor.

### Development of a *Hypophthalmichthys* genus-specific qPCR assay to quantify eDNA

Due to the high mitochondrial DNA (mtDNA) similarity between *H. nobilis* and *H. molitrix* [45] and their extensive hybridization in the Misssissippi basin [29], [30] we designed a *Hypophthalmichthys* genus-specific qPCR assay to exclusively measure the concentration of bigheaded carp eDNA in environmental samples. We designed a hydrolysis probe assay incorporating a Minor Groove-Binding (MGB) moiety to maximize specificity with minimal probe length [46], [47]. Primers and probes targeting only bigheaded carp were designed to maximize nucleotide mismatches between target and nontarget species using an alignment of all available mitochondrial control region (D-loop) sequences from NCBI GenBank for bigheaded carp and the most closely related species that potentially co-occur in North America: Common Carp (*Cyprinus carpio*), Goldfish (*Carassius auratus*), Grass Carp (*Ctenopharyngodon idella*), Black Carp (*Mylopharyngodon piceus*), and Crucian Carp (*Carassius carassius*). Melting temperature (T_m_) compatibility of oligonucleotides was checked using Primer Express v3.0.1 (Life Technologies, Carlsbad, California, USA). We conducted further *in silico* testing of species-specificity using the NCBI Primer-BLAST tool [48] and found no evidence of primer amplification outside the target genus.

**Figure 1:**
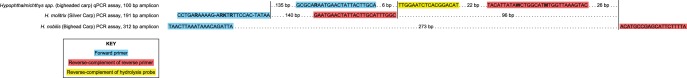
Alignment diagram showing the relative position of oligonucleotides for the qPCR and endpoint PCR assays. The qPCR assay was developed in the present study and the endpoint PCR assays are from a previous study [40]. Gaps (−) and bases in between oligonucleotides (… n bp…) reflect the actual alignment used to design the qPCR assay. Note that all oligonucleotides are shown on the ‘sense’ strand in 5′ to 3′ orientation, left to right. Thus oligonucleotides that actually bind to the sense strand (i.e., reverse primers and the hydrolysis probe) are shown as reverse-complements of the actual oligonucleotides used in an assay (Table 1). Degenerate bases are shown in bold text, but oligonucleotides were not synthesized with degenerate bases for the qPCR assay (Table 1). The forward primer of the Silver Carp endpoint PCR assay was synthesized with degenerate bases, as specified in the study that developed it [40].

A set of candidate primers and probes was selected for *in vitro* testing with 1 ng•µL^−1^ standardized tissue-derived total genomic DNA from *H. nobilis*, *H. molitrix*, *C. carpio*, *C. auratus*, and *C. idella*. Importantly, obtaining uncontaminated samples of tissue from these closely related, co-occurring species required considerable and repeated effort, and we recommend extreme care to prevent cross-contamination of assay validation samples during collection, handling, transport, storage, extraction, etc. qPCR assays targeting low level DNA are notoriously sensitive to contamination, and their use requires extra precautions that are uncommon in ecological research and even in standard genetics laboratories [49], [50]. We selected one primer set (100 bp amplicon) and one MGB probe (Table 1, Figure 1) that demonstrated identical amplification efficiency from both *H. nobilis* and *H. molitrix* DNA and no amplification from any of the nontarget species’ DNA. Note that we designed two slightly different versions of the forward and reverse primers (Table 1), reflecting three positions that vary within bigheaded carp, according to all available GenBank sequences. Rather than ordering primers with degenerate bases, which would create unnecessary combinations of the two variable positions in the reverse primer, we simply ordered each version separately and combined them at equimolar concentration. We also designed and tested a Locked Nucleic Acid (LNA; Sigma-Aldrich, St. Louis, Missouri, USA) [51] version of the probe (Table 1) and found equivalent performance (data not shown). All results in this study were generated using the MGB version of the probe.

**Table 1:** qPCR oligonucleotides developed for this study to produce a genus-specific assay targeting a 100 bp section of the D-loop mtDNA region in bigheaded carp (genus *Hypophthalmichthys*: *H. molitrix* and *H. nobilis*).

Type	Name	Sequence
forward primer	Hyp_Dlp_F_Hm	5′-GCG CA**G** AAT GAA CTA TTA CTT GCA-3′
forward primer	Hyp_Dlp_F_Hn	5′-GCG CA**A** AAT GAA CTA TTA CTT GCA-3′
reverse primer	Hyp_Dlp_R_Hm	5′-GTA CTT TAA CCA **G**AT GCC AG**A** TAT AAT GTA-3′
reverse primer	Hyp_Dlp_R_Hn	5′-GTA CTT TAA CCA **T**AT GCC AG**T** TAT AAT GTA-3′
hydrolysisMGB probe	Hyp_Dlp_MGB	5′-6FAM-ATG TCC GTG AGA TTC CAA-MGB-NFQ-3′
hydrolysisLNA probe	Hyp_Dlp_LNA	5′-6FAM-A+TG +TCC GT+G A+GA TT+C CAA-BHQ1-3′

Note that the two slightly different versions of the forward and reverse primers reflect three positions (shown in bold text) that vary within bigheaded carp, according to all available GenBank sequences. The “Hm” version of a primer matches all *H. molitrix* sequences while the “Hn” version matches all *H. nobilis* sequences in GenBank. Rather than ordering primers with degenerate bases, which would create unnecessary combinations of the two variable positions in the reverse primer, we simply ordered each version separately and combined them at equimolar concentration. The Hm and Hn variants do not provide discrimination between *H. molitrix* and *H. nobilis*. Rather, they are used in combination to maximize sensitivity within the genus while avoiding amplification from any non-*Hypophthalmichthys* species. 6FAM = fluorescein amidite reporter. MGB = minor groove binding moiety. NFQ = non-fluorescent quencher. BHQ1 = black hole quencher. +N = locked nucleic acid (LNA).

Finally, we conducted *in situ* testing of this assay using eDNA samples from various natural and manmade water bodies (Table S1). These included water bodies where bigheaded carp had previously been stocked or detected (target species present) and water bodies where neither bigheaded carp nor bigheaded carp eDNA had previously been detected (target species absent). For any site where at least one *in situ* testing eDNA sample produced at least one positive reaction with the new qPCR assay, we purified the qPCR product (ExoSAP-IT, USB/Affymetrix, Santa Clara, CA, USA) and submitted it to the University of Notre Dame Genomics Core Facility for bi-directional Sanger sequencing (ABI 3730xl, Applied Biosystems/Life Technologies, Foster City, CA, USA). Resulting chromatograms were quality trimmed by eye and taxonomically identified using alignment with the same set of sequences used to design the assay and with a standard nucleotide BLAST [52] search of the NCBI nr/nt database.

We performed all reactions on an Eppendorf Mastercycler ep realplex2 S thermocycler (Eppendorf, Hauppauge, New York, USA) with the following thermocycling conditions: 50°C for 2 min, 95°C for 10 min, and 55 two-step cycles of 95°C for 15 s and 60°C for 1 min. Fluorescence data collection occurred during the 60°C step. We used TaqMan Environmental Master Mix 2.0 (Life Technologies). We performed 20 µL triplicate reactions using 10 µL of commercial master mix, final primer concentrations of 300 nM each, a final probe concentration of 200 nM, and 4 µL of DNA extract. To minimize variation between replicate reactions caused by imperfect pipetting of small DNA extract volumes, we combined DNA extract and the final master mix (all other reagents) for three reactions into one tube then dispensed to three plate wells using an electronic repeating pipette (Xplorer 5–100 µL, Eppendorf) according to manufacturer recommended pipetting procedures. All liquid handling for qPCR used low bind tubes and low bind aerosol barrier pipette tips [53], and each qPCR plate included three no template control (NTC) reactions.

We used a copy number standard curve made by amplifying the entire mtDNA D-loop (1022 bp) [54] from tissue-derived *H. molitrix* DNA. After confirming a single gel electrophoresis band approximately 1000 bp in size, we purified this PCR amplicon using ExoSAP-IT (Affymetrix-USB Corporation, Santa Clara, California, USA) and quantified it using 5 µL of PCR product with a Qubit fluorometer and the Qubit dsDNA High Sensitivity kit (Life Technologies). We converted from DNA weight to DNA copies using the median double-stranded molecular weight of the 95% consensus 1022 bp amplicon sequence from all *H. molitrix* mitogenomes on GenBank (635518 g•mole^−1^) as calculated by OligoCalc [55]. This molecular weight calculation takes into account the actual base composition of the polynucleotide and yielded a weight equivalent to calculating molecular weight using an average weight of 618 Daltons•bp^−1^. We stored single-use 6.4×10^6^ copies•µL^−1^ (6.7×10^−3 ^ng•µL^−1^) aliquots of this standard DNA in low TE buffer (10 mM Tris, 0.1 mM EDTA) at −20°C. Five point qPCR standard curves were prepared by serial 10-fold dilution from 10^4^ copies•µL^−1^ down to 10^−1^ copies•µL^−1^ in low TE buffer and stored at 4°C while in use [56]. The fluorescence threshold for each plate and the fluorescence baseline for each reaction were determined using default settings of the Eppendorf realplex software version 2.2 (Noiseband and Automatic Baseline, respectively). Every amplification profile was visually examined to confirm exponential amplification. Following the recommendation of Ellison et al. [53] for qPCR with low level DNA, we calculated concentrations for each reaction, assigning zero concentration to non-detect reactions and averaging concentration across the three technical replicates for each eDNA extract.

### Comparison of methods

We compared our newly developed qPCR assay and eDNA capture/extraction methods with the existing endpoint PCR assays and eDNA capture/extraction methods for bigheaded carp eDNA surveillance using an experimental pond at the United States Geological Survey Columbia Environmental Research Center (USGS-CERC) in Columbia, Missouri, USA. USGS-CERC experimental pond 26, an earthen, gravel-lined pond measuring 37 m long by 21 m wide by 1 m deep and holding approximately 950 m^3^ of water, had previously been filled with well water and stocked with twelve juvenile Grass Carp (approximately 150–300 mm total length), five *H. nobilis* (approximately 200 mm total length), and one *H. molitrix* (approximately 200 mm total length). Over a five-day period (April 30, 2012– May 04, 2012) we conducted twelve sampling events at this pond. Each sampling event consisted of filling eight autoclaved 2-L bottles with surface water in paired fashion, two bottles filled simultaneously and side-by-side from each corner of the pond: one for GF filtration and one for PCTE filtration. A collection negative control bottle was filled with 2 L reverse osmosis (RO) water in the laboratory prior to sampling and was transported alongside sample bottles during collection for each sampling event.

eDNA capture by filtration was performed following Jerde et al. [40] except we placed filters in 300-mL single-use filter funnels (Pall 4815, Port Washington, New York, USA). We performed eDNA capture using GF 934-AH filters (1.5 µm nominal pore size, 47 mm diameter, Whatman) and PCTE filters (10 µm pore size, 47 mm diameter, GE Osmonics). For every sample, the full 2 L successfully passed through a single filter within approximately 10 min. After filtration we used bleach-decontaminated forceps to carefully fold the filter and place it into a 5-mL bead beating tube (GF filters) or a 2-mL microcentrifuge tube (PCTE filters). Negative controls were filtered alongside samples. Used filters were immediately frozen (−20°C), then transported to the University of Notre Dame on dry ice and stored in a −20°C freezer until eDNA extraction.

We extracted eDNA from GF filters with the PowerWater DNA Isolation Kit (MO BIO Laboratories, Carlsbad, California, USA) following Jerde et al. [40], but we included one extraction negative control with every batch of PowerWater DNA extractions and added pGEM-3Z plasmid (Promega, Madison, Wisconsin, USA) to Solution PW1 at 0.02 ng•µL^−1^ as internal positive control (IPC) DNA for PCR inhibition testing [44]. We extracted eDNA from PCTE filters using our modified CTAB protocol (Protocol S1), including one extraction negative control with every batch of extractions and pGEM-3Z plasmid in the CTAB buffer at 0.02 ng•µL^−1^ as IPC DNA for PCR inhibition testing [44]. Final eDNA pellets were re-suspended in 100 µL of low TE buffer. We tested all eDNA samples for PCR inhibition using the pGEM-specific IPC assay described in Coyne et al. [44], and we considered pGEM amplification as evidence for a lack of inhibition. All eDNA samples were assayed with the two endpoint PCR assays (one for *H. nobilis*, one for *H. molitrix*) following Jerde et al. [40] and with the bigheaded carp qPCR assay we developed. To compare detection probability (i.e., diagnostic sensitivity) between methods, we calculated the proportion of samples that tested positive and the associated 95% confidence interval for a binomial probability using the Wilson score method [57] and performed McNemar’s chi-squared (χ^2^) test for comparing proportions from paired data. To compare the bigheaded carp eDNA yield between capture/extraction methods we used Student’s paired t-test and the Wilcoxon signed-rank test. All statistical analyses used an alpha level of 0.05 and were performed in R version 3.0.1 [58] using R packages ‘stats’ version 3.0.1 and ‘binom’ version 1.1–1.

We also directly compared the analytical sensitivity of the three assays using serial dilutions of their respective purified amplicons to determine the 95% limit of detection (LOD) for each assay under ideal conditions (i.e., in the absence of any non-target DNA or other potential inhibitors). The lowest amplicon concentration that produced amplification in at least 95% of the technical replicates was considered the 95% LOD [59]. We used eight technical replicates for each endpoint PCR assay [40] and three technical replicates for the qPCR assay.

Finally, to test the hypothesis that sensitivity differences between assays were largely due to amplicon size and primer T_m_ agreement, we tested all three assays in one set of common reaction conditions. This required elimination of the hydrolysis probe for the *Hypophthalmichthys* genus-specific qPCR assay so that all assays could be tested with primers alone. Amplification was monitored in real time using SYBR Green I dye, which fluoresces upon binding to double-stranded DNA. Reaction conditions consisted of 25 µL total volume, 12.5 µL Platinum SYBR Green qPCR SuperMix-UDG (Invitrogen), primers at 0.2 µM each, and 1 µL template DNA. A 4.85×10^5^ copies•µL^−1^ solution of the entire mtDNA D-loop region [54], PCR-amplified and purified from *H. molitrix* or *H. nobilis* tissue-derived DNA, was used for template. Thermocycling conditions followed the manufacturer’s instructions for Platinum SYBR Green qPCR SuperMix-UDG and Platinum Taq (Invitrogen) and consisted of 50°C for 2 min, 95°C for 2 min, and 55 three-step cycles of 95°C for 30 s, 50°C for 30 s, and 72°C for 30 s. Fluorescence data collection occurred during the 72°C step. The *Hypophthalmichthys* genus-specific qPCR assay was separately tested with both *H. molitrix* template and *H. nobilis* template. The *H. molitrix* species-specific endpoint PCR assay was tested with *H. molitrix* template. The *H. nobilis* species-specific endpoint PCR assay was tested with *H. nobilis* template. All reactions were performed in triplicate on the same thermocycling run, allowing for calculation of a ΔCq value to compare assay sensitivity in one set of common reaction conditions. ΔCq was calculated as the average Cq of the triplicate reactions for a species-specific endpoint PCR assay minus the average Cq of the triplicate reactions for the genus-specific qPCR assay.

## Results and Discussion

### Improved assay for *Hypophthalmichthys* eDNA

Throughout the entire study, all collection negative controls, extraction negative controls, endpoint PCR NTCs, and qPCR NTCs tested negative. All eDNA samples passed the PCR inhibition test. *In silico*, *in vitro*, and *in situ* testing of the new bigheaded carp qPCR assay showed no evidence of amplification from DNA of non-target species (*in silico*: GenBank nr database; *in vitro*: *C. carpio*, *C. auratus* and *C. idella*; *in situ*: see Table S1), and Sanger sequencing of *in situ* testing samples confirmed amplification of the targeted 100 bp D-loop mtDNA amplicon from bigheaded carp (Table S1). Standard curves for all qPCR runs had r^2^≥0.98 and efficiency averaged 100%. The 95% LOD for the new bigheaded carp qPCR assay was 30 copies•reaction^−1^ (7 copies•µL^−1^ of amplicon solution) and the lowest concentration detected was 3 copies•reaction (1 copy•µL^−1^ of amplicon solution, 67% amplification). The 95% LOD for the old *H. nobilis* endpoint PCR assay was 2000 copies•reaction^−1^ (2000 copies•µL^−1^ of amplicon solution) and the lowest concentration detected was 200 copies•reaction^−1^ (200 copies•µL^−1^ of amplicon solution, 20% amplification). The 95% LOD for the old *H. molitrix* endpoint PCR assay was 200 copies•reaction^−1^ (200 copies•µL^−1^ of amplicon solution) and the lowest concentration detected was 10 copies•reaction^−1^ (10 copies•µL^−1^ of amplicon solution, 24% amplification).

In testing at the USGS-CERC experimental pond, the endpoint PCR assays collectively detected bigheaded carp eDNA in only 4 of 96 samples (two by the *H. nobilis* assay; two by the *H. molitrix* assay; 4.2% detection probability; Table 2). In contrast, the qPCR assay detected bigheaded carp eDNA in 91 of 96 samples (94.8% detection probability; Table 2). Thus the qPCR assay provided a 22-fold improvement in detection over the endpoint PCR assays (McNemar's χ^2^ = 85.0, df = 1, P<2.2×10^−16^).

**Table 2:** Detection probabilities of different methods for eDNA-based detection of bigheaded carp presence in an experimental pond containing six bigheaded carp.

Detectionmethod	Capturemethod	Extractionmethod	Detections;Non-detections	DetectionProbability (95% CI)
**qPCR assay**	10 µm PCTE	CTAB	46; 2	0.958 (0.860–0.988)
	1.5 µm GF	PowerWater	45; 3	0.938 (0.832–0.979)
	*Combined total*		*91; 5*	*0.948 (0.884–0.978)*
**endpoint PCR assays**	10 µm PCTE	CTAB	4; 44	0.083 (0.033–0.196)
	1.5 µm GF	PowerWater	0; 48	0 (0–0.074)
	*Combined total*		*4; 92*	*0.042 (0.016–0.102)*

PCTE = polycarbonate track-etched filter membrane. GF = glass fiber filter paper. CTAB = cetyl trimethyl ammonium bromide DNA extraction protocol. PowerWater = PowerWater DNA Isolation Kit, MO BIO Laboratories.

The new qPCR assay was designed for eDNA quantification using real-time fluorescent detection of amplification and hydrolysis probe chemistry, not by optimizing from the old endpoint PCR assays. Its design therefore followed the guidelines and practices of real-time quantitative PCR for low-level DNA in environmental mixtures including amplicon size <150 bp, primer melting temperatures near 60°C, inclusion of a probe, and use of inhibitor-resistant reagents [60]. These features prevent precise determination of the mechanisms producing the 22-fold higher sensitivity of the qPCR assay compared to the endpoint PCR assays. In fact, the kinetics of primer- and probe-template hybridization create idiosyncrasies that cannot be completely controlled for when comparing performance between PCR assays [60]. Our objective was not to determine what makes one assay more sensitive than another, but rather to provide the first quantitative assay for bigheaded carp eDNA and compare it against the assays currently being used to monitor this invasive species.

Nevertheless, three differences between the new qPCR assay and the old endpoint PCR assays stand out as likely contributors to the 22-fold sensitivity difference we observed: amount of DNA per reaction, amplicon size, and primer T_m_ agreement [60]. The qPCR assay used 4 µL of DNA in 20 µL reactions while the PCR assays used 1 µL of DNA in 25 µL reactions - a 5-fold difference in template DNA concentration. The qPCR assay amplifies a 100 bp amplicon while the PCR assays amplify 191 bp and 312 bp amplicons, respectively. Lastly, the T_m_ differences are 0.3°C for the qPCR primers, 0°C for the *H. molitrix* PCR primers, and 15.2°C for the *H. nobilis* PCR primers. The effects of amplicon size and primer T_m_ could not be separated because primer sequence determines both. We therefore performed a laboratory comparison of all three assays under identical reaction conditions to isolate the effect of amplicon size and primer T_m_. We calculated the ΔCq between each endpoint PCR assay and the qPCR assay using identical starting concentrations of template DNA. ΔCq for the *H. molitrix* endpoint PCR assay compared to the qPCR assay was 8.7 cycles. ΔCq for the *H. nobilis* endpoint PCR assay compared to the qPCR assay was 16.3 cycles. Thus with all else held constant, the *H. molitrix* endpoint PCR primers reached the exponential amplification phase 8.7 cycles later than the *Hypophthalmichthys* qPCR primers. For the *H. nobilis* primers this phase occurred 16.3 cycles later. These amplification delays demonstrate that the greater sensitivity of the *Hypophthalmichthys* qPCR assay is largely due to its small amplicon size and close primer T_m_ agreement. Thus it would be incorrect to assume that qPCR technology (i.e., real-time fluorescent detection of amplification), in itself, provided the increase in sensitivity over endpoint PCR technology (i.e., endpoint fluorescent detection by gel electrophoresis). Overall, the 22-fold greater sensitivity of the new qPCR assay described here is sufficient to recommend its replacement of the old endpoint PCR assays for eDNA-based monitoring of bigheaded carp.

The low detection probability of the endpoint PCR assays (Table 2) was surprising given their successful field application in areas where bigheaded carp are rare [25], [40]. We ruled out PCR inhibition as the cause by using the pGEM IPC assay and by diluting samples and re-assaying them with the endpoint PCR assays. qPCR assay of the same samples ruled out failure to capture or recover bigheaded carp eDNA (Table 2) but demonstrated that its concentration was generally too low for detection by the endpoint PCR assays. The concentration of qPCR-measured bigheaded carp eDNA in the eDNA extracts ranged from 1 to 161 copies•µL^−1^ of eDNA extract overall, and from 1 to 26 copies•µL^−1^ of eDNA extract in the four samples producing endpoint PCR detection. The endpoint PCR assays specify 1 µL of eDNA extract per 25 µL reaction [40], thus the starting template concentration was always below the 95% LOD of either endpoint PCR assay (2000 and 200 copies•µL^−1^ of eDNA extract, respectively). The larger amplicons of the endpoint PCR assays likely further reduced template concentration relative to the qPCR assay, because shorter fragments of a given DNA region are more abundant in environmental samples [61]. In summary, the low detection probability we observed for the endpoint PCR assays is consistent with their analytical sensitivity given the low concentration of qPCR-measured bigheaded carp eDNA in the experimental pond. This result suggests that recent surveys may have failed to detect bigheaded carp eDNA when it was actually present.

### Improved eDNA capture and extraction methods

The advantage of eDNA-based monitoring over direct observation rests primarily on increased sensitivity and/or cost-effectiveness [19]. This principle led us to make improvements on the methods developed by Jerde et al. [40], which are currently the standard operating procedures for eDNA surveillance in the ongoing binational effort to prevent bigheaded carp from establishing in the Great Lakes [27], [62]. Those eDNA capture and extraction methods present several opportunities for increasing sensitivity and decreasing cost. The chosen glass fiber (GF) filter (934-AH, Whatman) is only available in a small range of nominal pore sizes (0.7–2.7 µm) and is poorly suited for subsequent DNA extraction. Its use in the PowerWater DNA Isolation Kit requires substantial deviation from the manufacturer’s recommendation to use only one carefully rolled filter per extraction tube and to perform bead beating for only 5 min. The protocol from Jerde et al. [25], [40] packs up to four filters in the extraction tube and performs bead beating until the filters are ground into a slurry, sometimes requiring more than 60 min of bead beating (C.R.T, pers. obs.). The long bead beating step costs researcher time and increases the risk of DNA shearing [63], yet bead beating a single GF filter for only 5 min can fail to yield any target DNA, even when it was captured in abundance (C.R.T., unpublished data). In addition to the costs incurred by the long duration of this extraction, its purchase price is also high ($8.32 USD per extraction). When particularly turbid waters require more than eight filters to process the standard 2 L sample, the extraction consumables cost for one sample reaches $25 USD.

In contrast, Coyne et al. [43], [44] describe the use of a non-commercial CTAB DNA extraction protocol [64] for recovering aqueous microbial eDNA from filters. The CTAB protocol uses chloroform to chemically dissolve PCTE filters during the extraction, simplifying DNA recovery and allowing easy scaling of the extraction volume to accommodate many filters if needed. CTAB DNA extraction is widely used because of its versatility [43], high DNA yield [65], effective inhibitor removal [66], and extremely low cost (approximately $0.20 USD per 2 mL extraction). For these reasons, we applied the protocol of Coyne et al. [43], [44] to extract aqueous macrobial eDNA from PCTE filters, which are available in a wide range of pore sizes (0.1–20 µm; GE Osmonics, sold by Barney Corporation, Hilliard, Ohio, USA) and primarily retain particles on the filter surface rather than deeply embedded in the filter matrix like glass fiber and cellulosic filters [67], [68].

**Figure 2:**
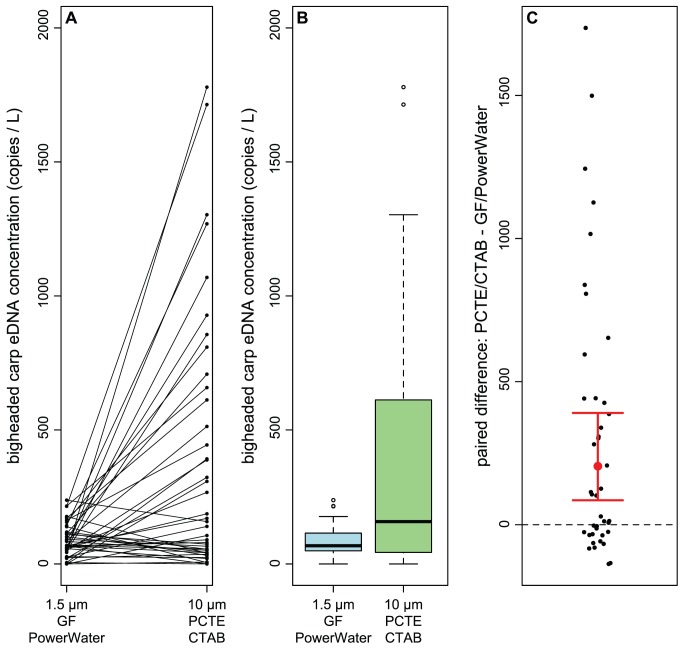
qPCR assay results comparing the amount of bigheaded carp eDNA captured and recovered (i.e., eDNA yield) using two alternative capture/extraction methods on paired 2 L samples collected side-by-side in the experimental pond at USGS-CERC. 48 pairs of samples were collected, but following Zuur et al. [88], seven pairs with unusually high eDNA concentration (i.e., outliers) were removed prior to statistical analysis. Statistical results were robust to outlier presence or removal, and plots including outliers are provided in Figure S1. PCTE = polycarbonate track-etched filter membrane, GF = glass fiber filter paper, CTAB = cetyl trimethyl ammonium bromide DNA extraction protocol, PowerWater = PowerWater DNA Isolation Kit. (A) Paired data, (B) boxplot, (C) paired differences and the median difference (red point) and 95% confidence interval (red interval) from the Wilcoxon signed-rank test. Note that points in (C) are horizontally ‘jittered’ for better visualization. The PCTE/CTAB method yielded significantly more eDNA than the GF/PowerWater method (paired Student’s t-test, t = 4.1, df = 40, P = 0.00019).

In testing at the USGS-CERC experimental pond, detection probability was not significantly different between eDNA capture/extraction methods for either type of assay (endpoint PCR or qPCR; McNemar's χ^2^ = 2.25, df = 1, P = 0.13, McNemar's χ^2^ = 0, df = 1, P = 1; Table 2) but the PCTE filter and CTAB extraction method yielded five times more bigheaded carp eDNA, on average, than the GF filter and PowerWater extraction (paired Student’s t-test, t = 4.1, df = 40, P = 0.00019; Figure 2). The 5-fold higher yield may reflect higher eDNA capture, extraction recovery, or both. However, the larger effective [69], [70] and nominal pore size of the PCTE filter (10 µm vs. 1.5 µm for GF) suggests eDNA capture would be higher for the GF filter. This points to extraction recovery as the more important process influencing final yield in our comparison, which is expected because phase separation and precipitation methods (e.g., CTAB) consistently yield more DNA than silica column methods (e.g., PowerWater) [71], [72]. Thus we recommend the CTAB extraction method for general application in eDNA-based monitoring. Its efficacy has been demonstrated on a wide range of environmental samples, from aquatic sediments [43], [73] and volcanic rock [74] to retentate on various types of filter material including PCTE [44], polyethersulfone (PES) [75], cellulose nitrate (nitrocellulose, pyroxylin) [76], cellulose acetate [77], glass fiber [78], and nylon net [79]. During the CTAB extraction, chloroform dissolves PCTE [80] and PES filters [81], facilitating the recovery of any DNA-containing particles embedded in the filter matrix [67], [82]. This chemical dissolution also enables simple scale up of the extraction volume to accommodate multiple filters or large surface area filters. Finally, for eDNA capture in the field, immediately storing a used filter in CTAB buffer (Protocol S1) reduces DNA degradation [83]–[85] because CTAB lyses cells while EDTA and salt inactivate nucleases.

### Conclusions

In testing at the USGS-CERC experimental pond, the standard procedure for detecting bigheaded carp eDNA (Table 3) failed to produce a single positive detection with either endpoint PCR assay, even though the pond contained one *H. molitrix* and five *H. nobilis* (Table 2). In contrast, our new procedure (Table 3) detected bigheaded carp presence with 95.8% detection probability (McNemar's χ^2^ = 44.0, df = 1, P = 3.2×10^−11^; Table 2). The new qPCR assay described here provided a 22-fold higher detection probability than the standard endpoint PCR assays, and the new eDNA capture and extraction method yielded five times more bigheaded carp eDNA than the standard method (Figure 2), at a per sample extraction cost over forty times lower.

**Table 3:** Comparison of existing and new protocols for eDNA-based monitoring of bigheaded carp.

Step	Standard Procedure [40]	New Procedure (this study)
**1. Sample Collection**	2-L bottle filled with surface water	Same
**2. eDNA Capture**	Filtration with glass fiber filter(Whatman grade 934-AH, 1.5 µmnominal pore size, 47 mm diameter)	Filtration with polycarbonate filter (GE Osmonics,10 µm nominal pore size, 47 mm diameter)
**3. eDNA Extraction**	MO BIO PowerWaterDNA Isolation Kit	CTAB extraction
	Lengthy bead beatingcompletely grinds filters	Chloroform rapidlydissolves filters
	No extraction negativecontrols	Extraction negativecontrols with every batch
**4. eDNA Assay**	Two endpointPCR assays	One qPCR assay
	Assays targetmtDNA ‘species’	Assay targets genus
	191 and 312 bpamplicons	100 bp amplicon
	primers providespecificity	primers and probeprovide specificity
	1 µL of DNA in25 µL reaction	4 µL of DNA in 20 µL reaction
	8 technical replicatesper assay	3 technical replicates
	3-step thermocyclingprogram	2-step thermocycling program
	45 thermocycles	55 thermocycles*
	Gel electrophoresisvisual detection	Real-time fluorescence detection

*The latest Cq observed was in cycle 41.

The standard operating procedure for eDNA-based monitoring of bigheaded carp regularly detects *H. molitrix* eDNA, and occasionally *H. nobilis* eDNA, in surveillance of invasion pathways to the Great Lakes [27], [28], but in this study it failed to detect one *H. molitrix* and five *H. nobilis* in a 0.08 hectare pond (Table 2). This unexpected result suggests that bigheaded carp eDNA concentration is often higher in these invasion pathways than it was in the pond. Perhaps the bigheaded carp in these invasion pathways (e.g., the Chicago Area Waterway System) are larger or more abundant than they were in the pond. Another possibility is that environmental conditions in those lotic rivers and canals create aggregations of bigheaded carp eDNA not found in the lentic pond [86], [87]. Whatever the cause may be, our new qPCR assay for eDNA-based monitoring of bigheaded carp will increase detection probability compared to the current assays, while removing the contamination risk, subjectivity, cost, and time required for gel electrophoresis. Quantitative data from qPCR may also help establish minimum thresholds of eDNA concentration for reliably inferring recent and local bigheaded carp presence, and allow investigation of the relationship between bigheaded carp abundance and eDNA concentration.

## Supporting Information

Figure S1 **qPCR assay results, including outliers excluded from** **Figure 2** **, comparing the amount of bigheaded carp eDNA captured and recovered (i.e., eDNA yield) using two alternative capture/extraction methods on paired 2 L samples collected side-by-side in the experimental pond at USGS-CERC.** PCTE = polycarbonate track-etched filter membrane, GF = glass fiber filter paper, CTAB = cetyl trimethyl ammonium bromide DNA extraction protocol, PowerWater = PowerWater DNA Isolation Kit. (A) Paired data, (B) boxplot, (C) paired differences and the median difference (red point) and 95% confidence interval (red interval) from the Wilcoxon signed-rank test. Note that points in (C) are horizontally ‘jittered’ for better visualization. N = 48 pairs of samples. The PCTE/CTAB method yielded significantly more eDNA than the GF/PowerWater method (paired Student’s t-test, t = 2.87, df = 47, P = 0.006).
(EPS)

Table S1 ***In situ*** **specificity testing of the bigheaded carp qPCR assay.**
(DOCX)

Protocol S1 **CTAB DNA extraction protocol for PCTE or PES filters.**
(DOCX)
