# Supplementary material for: Improved Methods for Capture, Extraction, and Quantitative Assay of Environmental DNA from Asian Bigheaded Carp (Hypophthalmichthys spp.)
Source: PLoS One. 2014 Dec 4;9(12):e114329. doi: 10.1371/journal.pone.0114329 (PMC4256254; doi:10.1371/journal.pone.0114329)
Supplement: Table S1 — In situ specificity testing of the bigheaded carp qPCR assay. (DOCX) [file pone.0114329.s002.docx]

**Supporting Table S1**. *In situ* specificity testing of the bigheaded carp qPCR assay.

| **eDNA source** | **Public or private land** | **Permission or permit required** | **Location (LatDD; LonDD)** | **eDNA sample type and DNA extraction** | **Bigheaded carp occurrence** | **Non-target fish occurrence** | **qPCR result** | **Amplicon sequencing result** |
| --- | --- | --- | --- | --- | --- | --- | --- | --- |
| Potawatomi Zoo pond, South Bend, IN, USA | private | permission from Zoo director | 41.670629;  -86.216634 | aqueous, PCTE/CTAB | no | Common Carp | negative | n/a |
| St. Mary’s Lake, Notre Dame, IN, USA | private | permission from U. Notre Dame security police | 41.701497;  -86.244312 | aqueous, PCTE/CTAB | no | ~10 species | negative | n/a |
| St. Joseph River, South Bend, IN, USA | public | none required | 41.687419;  -86.252882 | aqueous, PCTE/CTAB | no | ~84 species | negative | n/a |
| USGS-CERC pond 26, Columbia, MO, USA | private | permission from USGS-CERC director | 38.911961;  -92.276837 | aqueous, PCTE/CTAB and GF/PowerWater | 5 *H. nobilis* and 1 *H. molitrix* | Grass Carp | positive | all sequences matched bigheaded carp |
| USGS-CERC ponds 27, 28, 30, Columbia, MO, USA | private | permission from USGS-CERC director | 38.911961;  -92.276837 | sedimentary, PowerSoil (MO BIO Laboratories) | previously contained *H. nobilis* and *H. molitrix* | previously contained Grass Carp and Common Carp | positive | all sequences matched bigheaded carp |
| KUFS ponds (10), Lawrence, KS, USA | private | permission from KUFS director | 39.047452; -95.191526 | aqueous/PCTE/CTAB and sedimentary/CTAB | *H. nobilis* and/or *H. molitrix* | Common Carp, Grass Carp, White Crappie, Bluegill Sunfish, and Redear Sunfish | positive | all sequences matched bigheaded carp |
| Wabash River, IN, USA | public | none required | 40.412487;  -87.036364 | aqueous/PCTE/CTAB and sedimentary/CTAB | *H. nobilis* and *H. molitrix* | ~150 species | positive | all sequences matched bigheaded carp |
