# Supplementary material for: Improved Methods for Capture, Extraction, and Quantitative Assay of Environmental DNA from Asian Bigheaded Carp (Hypophthalmichthys spp.)
Source: PLoS One. 2014 Dec 4;9(12):e114329. doi: 10.1371/journal.pone.0114329 (PMC4256254; doi:10.1371/journal.pone.0114329)
Supplement: Protocol S1 — CTAB DNA extraction protocol for PCTE or PES filters. (DOCX) [file pone.0114329.s003.docx]

**Supporting Protocol S1**. CTAB DNA extraction protocol for PCTE or PES filters.

Modified from Dempster et al. 1999, Coyne et al. 2001, and Coyne et al. 2005 (see main text).

Membrane preservation

1. Place the filter membrane in a contamination-free, 2 mL, solvent-resistant, microcentrifuge tube containing 700 µL of CTAB extraction buffer and store in a freezer.

Preparations for DNA extraction

1. Decontaminate the bench surface where you will be working (lab bench and fume hood) with 10% bleach and/or UV irradiation.
2. Decontaminate the equipment you will be using (pipettes, vortexers, centrifuges, etc.) with 10% bleach, DNA Away (Thermo Scientific) and/or UV irradiation.
3. Ensure that you have sufficient supplies:
   - Contamination-free, single-user aliquots of all reagents.
   - Contamination-free pipette tips (filter tips).
   - Contamination-free 1.5 mL, low adhesion microcentrifuge tubes.
4. Include one extraction negative control with every batch of extractions.

DNA Extraction

1. Heat samples in a 65ºC water bath for 10 min.
2. Cool briefly, then add 700 µL of chloroform:isoamyl alcohol (24:1) (Sevag; do this inside the fume hood).
3. Vortex the sample tubes and then shake at low speed for 5 min with the tubes in vertical position, to dissolve the filter and lyse cells (do this on a shaker in the fume hood).
4. Centrifuge at 15,000x g for 15 min at room temperature to separate the solvent phase, non-DNA solids, and aqueous phase (now containing dissolved DNA).
5. Using a 1000 µL pipette, carefully pipette all of the supernatant (aqueous phase, usually ~500 µL) to a new 1.5 mL low adhesion microtube, avoiding any subnatant or intermediate material.

DNA Precipitation

1. Add an equal volume (~500 µL) of ice-cold isopropanol.
2. Add a half volume (~250 µL) of 5 M NaCl.
3. Incubate at -20ºC for ~1 hour.
4. Centrifuge at 15,000x g for 15 min at room temperature. DNA will form a small pellet adhered to the bottom of the tube, but it may not be visible.
5. Carefully pour off the supernatant without losing the DNA pellet. If no pellet is visible just pour slowly and gently.
6. Add 150 µL of 70% ethanol, washing down the inner walls of the tube.
7. Centrifuge at 15,000x g for 5 min, then carefully pour or pipette off the ethanol.
8. Repeat steps 6 and 7 once.
9. Remove residual ethanol by air drying or use a Vacufuge (45ºC, V-AL, 5-10 min). Be careful not to overdry/cook the DNA pellet!
10. Resuspend the pellet in 100 µL of 60ºC warmed low TE buffer. Heat for 10 min in a 55ºC water bath then vortex gently. If necessary, you can speed re-suspension by gently mixing the liquid by pipetting with a low adhesion filter tip.
11. Store the re-suspended DNA in a 4ºC refrigerator to await genetic assay.

PCR Inhibitor Removal (optional)

1. If desired, the final eDNA extract can be further cleaned using the OneStep PCR Inhibitor Removal Kit (Zymo Research, Irvine, California, USA). If you will add this step we recommend resuspending the eDNA pellet in 200 µL of low TE buffer for maximum compatibility with the OneStep kit protocol.

**Recipes for eDNA extraction solutions**

General reminders:

- Use glass media bottles that have been cleaned, decontaminated with 10% bleach, rinsed with reverse osmosis (RO) water, and autoclaved.
- Decontaminate magnetic stir bars and the stir bar retriever by bleaching and rinsing with autoclaved RO water before every use.
- Decontaminate the pH sensor by bleaching and rinsing with autoclaved RO water before every use.

CTAB preservation/extraction buffer

--- This buffer should not be autoclaved so use extreme precautions against contamination. ---

- 100 mM Tris-HCl
  - - - - *For 1 Liter → 100 mL of 1M Tris-HCl (pH 8.0)*
- 1.4 M NaCl
  - - - - *For 1 Liter → 280 mL of 5M NaCl solution*
- 1% (wt./vol.) polyvinylpyrrolidone (PVP; molecular weight 360,000)
  - - - - *For 1 Liter → 10 g of PVP powder*
- 2% (wt./vol.) cetyl trimethyl ammonium bromide (CTAB)
  - - - - *For 1 Liter → 20 g of CTAB powder*
- 20 mM EDTA
  - - - - *For 1 Liter → 40 mL of 0.5M EDTA solution (pH 8.0)*
- Bring to a total volume of 1 Liter using autoclaved, RO water.
- Heat and stir at a low temperature until all solids have dissolved.
- Store at room temperature for up to 6 months.
- Make single-user and/or single-project aliquots as needed.
- Add pGEM-3Z plasmid (or another IPC DNA) at the desired concentration.

5M NaCl solution

- Use solid NaCl.
- Bring to final volume using RO water.
- Autoclave on liquid setting.

Low TE buffer (pH 8)

- 10mM Tris (use 1M Tris-HCl).
- 0.1mM EDTA (use 0.5M EDTA).
- Bring to final volume using RO water.
- Check pH and adjust using HCl, as needed.
- Autoclave on liquid setting.

Components that can be purchased pre-made

- Sevag (24:1 chloroform:isoamyl alcohol) - store in fume hood protected from light
- 100% Ethanol
- Isopropanol
- Low TE buffer
- 5M NaCl solution
- 1M Tris-HCl solution
- 0.5M EDTA solution
- PVP solid, molecular weight 360,000
- CTAB solid
- pGEM-3Z plasmid (Promega catalog # P2151)
